# A narrative review of the quality of life scales specific for chronic venous diseases

**DOI:** 10.1097/MD.0000000000025921

**Published:** 2021-05-21

**Authors:** Zhoupeng Wu, Yukui Ma

**Affiliations:** Department of Vascular Surgery, West China Hospital, Sichuan University, Chengdu, China.

**Keywords:** chronic venous disease, quality of life, scale

## Abstract

To review the various quality of life (QoL) scales specific for chronic venous diseases (CVDs) and provide guidance and a reference for researchers to select the ideal measurement scale before studying QoL in patients with CVDs.

The EBSCO, Ovid, PubMed, Wanfang, and CNKI databases were searched for the keywords “vein,” “quality of life,” and “scale/questionnaire,” and various scales used to measure QoL in patients with CVDs. The QoL aspects were investigated and researched, and then, the search results were screened and summarized.

A total of 10 major scales related to the QoL in patients with CVDs were included. The scales differed in dimension, reliability, validity, scoring method, evaluation method, and scope of application.

The investigator should select the chronic venous disease QoL scale according to the research purpose and subjects, and then implement the scale to compare the specific aspects of QoL in patients with different CVDs.

## 1 Introduction

Chronic venous disease (CVD) refers to a series of signs and symptoms caused by poor venous return and excessive venous pressure due to abnormal vein structure and function. CVD is more extensive than chronic venous insufficiency, which includes mild telangiectasia, spider veins, and reticular veins; however, they are often confused with each other in clinical practice. According to several epidemiological surveys,^[1–4]^ the incidence of venous diseases of lower extremities in Europe and the United States of 19% to 70% is higher than the incidence of CVD among the general Chinese population.^[5]^ Only a survey conducted in East China in 1988 reported the prevalence of peripheral vascular disease in the general population to be as low as 8.9%.^[6]^ With changes in lifestyle and advances in medical technology in recent years, the annual incidence of CVD has increased, and the changes in symptoms, activities, and quality of life (QoL) in patients with CVD have received increasing attention from researchers.

Various scales have been designed and used to assess the QoL in patients with CVD. It is imperative to understand the differences in the characteristics, scope, and application of each scale so that reliable and accurate results are reported in the context of the research aim. Therefore, our review intended to inform future researchers about the most commonly used CVD QoL scales.

## 2 Materials and methods

### 2.1 Scale search method

The China National Knowledge Infrastructure (CNKI) database and the Wanfang Data Knowledge Service Platform were searched for the keywords “venous,” “quality of life,” “scale/questionnaire,” and “venous/vein.” The terms “quality of life” and “instrument/scale/questionnaire” were searched for in EBSCO, Ovid, and PubMed databases.

### 2.2 Scale inclusion and exclusion criteria

Scale inclusion criteria were as follows: the scale has been used in quantitative studies, in the study of the QoL in patients with CVD, and includes basic information on the performance of the research tool.

### 2.3 Scale screening

Two researchers conducted a preliminary screening and evaluation of the Chinese and English abstracts of the retrieved documents, selected the literature involving the use of the CVD QoL scale, conducted cross-checking, and selected the scales that fulfilled the inclusion and exclusion criteria.

## 3 Results

### 3.1 Basic information on the included QoL scales specific for CVDs

After the literature search and screening, a total of 10 scales were included, which can be mainly divided into universal and specific scales.^[7–17]^ The basic information on each scale is shown in Table 1.

**Table 1:** Basic information on the quality of life in chronic venous diseases.

Scale	Researcher or institution	Year	Fill in the time	Applicable situation
SF-36	Boston New England Medical Center Health Institute ^[7]^	1993	No report	Universal
EQ-5D	European Quality of Life Study Group ^[8]^	1999	No report	Universal
AVVQ	Garratt AM^[9]^	1993	No report	Specificity
CIVIQ-20	R. Launois^[10]^	1996	No report	Specificity
VEINES-QoL/Sym	Lamping DL^[11–14]^	1998	10 min	Specificity
CCVLUQ	Smith JJ^[15]^	2000	No report	Specificity
DVTQOL	Ewa Hedner^[16]^	2004	No report	Specificity
VLU-QoL	Hareendran^[17]^	2007	No report	Specificity
SPVLU-5D	Palfreyman^[18]^	2008	No report	Specificity
CIVIQ-14	Tamara Sinožić^[19]^	2017	No report	Specificity

### 3.2 Psychometric indicators of the QoL in patients with CVD

The comparison of the psychometric indicators of the QoL in patients with CVD is shown in Table 2. The scale's reliability was predominantly evaluated using Cronbach's α coefficient and the reproducibility of the test–retest method. Validity was measured by the content validity index, exploratory factor analysis, and correlation analysis. Each scale had good reliability in empirical research. Cronbach's α coefficient was above 0.72, and the test–retest reliability was between 0.66 and 0.98. A detailed report on the validity of the European Quality of Life—5 Dimensions (EQ-5D) and the Sheffield Preference-based Venous Ulcer questionnaire—5 Dimensions (SPVLU-5D) scales was not found. Some scales that used the Short Form 36-Item (SF-36) questionnaire as the calibration, such as the Aberdeen Varicose Vein Questionnaire (AVVQ), the Charing Cross Venous Leg Ulcer Questionnaire (CCVLUQ), and the Venous Leg Ulcer Quality of Life (VLU-QoL) questionnaire, showed higher correlation and better content validity.

**Table 2:** Life quality scale's psychometric indicators of chronic venous disease.

Scale	Reliability	Validity
SF-36	Cronbach's α is 0.72–0.88, and the retest reliability is 0.66–0.94.^[21]^	Factor analysis produces 2 principal components with a cumulative variance contribution rate of 56.3% and good validity.^[21]^
EQ-5D	Cronbach's α is 0.89, and the retest reliability is 0.93.^[23]^	No report
AVVQ	Cronbach's α is 0.72–0.76, and the retest reliability is 0.86.^[9,20,24]^	Exploratory factor analysis extracts 6 common factors and ultimately retains 4 common factors. Compared with SF-36, it has higher correlation and better validity.^[20]^
CIVIQ-20	Cronbach's α is 0.65–0.90, and the retest reliability is 0.85–0.98.^[10]^	The factor analysis has good structural validity and the variance interpretation is 46.9%.^[10]^
VEINES-QoL/Sym	Cronbach's α is 0.88–0.91, and the retest reliability is 0.87.^[25]^	Four common factors were extracted and named as symptoms, physiological functions, physiological capacity, mental health, cumulative variance contribution rate of 69.85%, S-CVI of 0.97, and I-CVI of 0.8–1.00 for each questionnaire.^[25]^
CCVLUQ	Total Cronbach's α is 0.93, and the retest reliability is 0.84.^[25]^	Factor analysis extracts 7 common factors, and retains 4 common factors according to expert opinions. The load factor of each item is between 0.40 and 0.83. With SF-36 as the calibration, the correlation coefficient *r* is 0.32–0.70.^[25]^
DVTQOL	Cronbach's α is 0.79–0.93.^[8]^	No report
VLU-QoL	Cronbach's α > 0.80 in each dimension, and the retest reliability *r* value is 0.83–0.86.^[17]^	Exploratory factor analysis extracted 3 common factors, each item factor load coefficient was greater than 0.4; with SF-36 as the calibration, the correlation coefficient *r* value was 0.46–0.64^[17]^
SPVLU-5D	Cronbach's α is 0.93.^[18]^	No report
CIVIQ-14	The total Cronbach's α is 0.92, the Cronbach's α ≥ 0.80 in each dimension, and the average correlation coefficient between the scale items is 0.45, and the dimensions are 0.45–0.67, respectively.^[19]^	The factor analysis has good structural validity, KMO = 0.93, and the variance interpretation is 59%.^[19]^

### 3.3 Dimensional content and scoring method of the QoL in patients with CVD

For the QoL scales used in patients with CVDs and included in this study, the number of dimensions ranged from 2 to 8, and the number of entries was 6 to 36. The presentation of the disease was mainly based on the symptoms of the disease, and the physiological, psychological, and social aspects of the treatment process. The measurement methods were all self-assessed by patients. The multi-quantity table lacked the report of filling time. SF-36 and EQ-5D are widely used in patients with CVDs and are often investigated in parallel with the CVD-specific scale in the same population.^[20,21]^ The correlation between the scales is investigated to verify the validity of the specific scale for patients with CVDs. The measurement contents of the scales are different, but there are also some associations. For example, the ChronIc Venous Insufficiency Questionnaire (CIVIQ) was originally developed based on SF-36^[10]^; thus, it is easy to see that the division of dimensions by CIVIQ draws SF-36 theoretical basis to some extent. The specific measurement dimensions and scoring methods of the QoL indicators for CVDs are shown in Table 3.

**Table 3:** Quality of life scale's dimensional content and scoring method of each chronic venous disease.

Scales	Dimensions	Items	Dimensional contents	Scoring method
SF-36	8	36	Physiological function. Physiological capacity. Somatic pain. General health condition. Energy. Social function. Emotional function. Mental health.	Likert degree 5
EQ-5D	5	6	Activity. Self-care. Daily activity. Pain or comfortlessness. Anxiety or depression.	It is divided into 2 parts. The part designed to describe health condition adopt Likert degree 3. The part of VAS adopts percentile system.
AVVQ	4	13	Pain or functional disorder. Appearance. Severity. Complications.	Likert degree 2–4
CIVIQ-20	4	20	Body. Psychology. Society. Pain.	Likert degree 5
VEINES-QoL/Sym	2	26	Life quality. Symptom	Likert degree 2–6
CCVLUQ	4	20	Social activity. Daily activity. Appearance. Mood.	Likert degree5
DVTQOL	6	29	Mental disorder. Symptom. Activity limit. Anticoagulation management. Sleep disorder. Diet problem.	Likert degree 7
VLU-QoL	3	34	Daily life effect. Subjective feeling effect. Local symptom.	Likert degree 5
SPVLU-5D	5	16	Pain. Activity. Emotion. Ulcerative odour. Social activity.	Likert degree 5
CIVIQ-14	3	14	Body. Psychology. Pain.	Likert degree 5

### 3.4 Comparison of the contents of the QoL scales in patients with CVD and their advantages and disadvantages

This study included 10 scales covering the aspects of pain, physical activity, daily life, appearance, social activity, mood, sleep, medication, diet, self-care, protective measures, complications, and ulcers, for patients with CVDs. The contents of each QoL scale and its advantages and disadvantages are compared in Tables 4 and 5.

**Table 4:** Comparisons of scales that appraisal chronic venous diseases’ life quality.

Rating scales	Pain	Physical activity	Daily life	Appearance	Social activity	Emotion	Sleep	Medication	Diet	Self-care	Protective measures	Complications	Ulcer condition
SF-36	√	√	√	—	√	√	—	—	—	—	—	—	—
EQ-5D	√	√	√	—	—	√	—	—	—	√	—	—	—
AVVQ	√	—	√	√	—	—	—	√	—	—	√	√	√
CIVIQ-20	√	√	√	√	√	√	√	—	—	—	√	—	—
VEINES-QoL/Sym	√	√	√	√	√	√	—	—	—	—	—	—	—
CCVLUQ	√	√	√	√	√	√	—	—	—	—	—	—	√
DVTQOL	√	√	—	—	—	√	√	√	√	—	—	—	—
VLU-QoL	√	√	√	√	√	√	√	—	—	√	—	—	√
SPVLU-5D	√	√	—	—	√	√	—	—	—	—	—	—	√
CIVIQ-14	√	√	√	√	√	√	√	—	—	—	—	—	—

**Table 5:** Advantages and disadvantages of each rating scale designed to assess chronic venous diseases’ life quality.

Scales	Advantages	Disadvantages
SF-36	It is widely used, and can be compared with the results of other diseases.	It is time-consuming and has plenty of items and does not involve specific content.
EQ-5D	It is divided into 2 parts: health description system and EQ-VAS, and can be filled in easily.	The items are not specific and has not been reported used for CVD patients.
AVVQ	The first question requires the patient to draw the position of his own venous lesion, which can clarify the location of the lesion. It is a scale specifically designed for patients with varicose veins.	It does not involve social activities, emotions, and sleeps.
CIVIQ-20	It has comprehensive content, good reliability, and it is widely used.	Some studies thought its structure was unstable and some item had not entered the structure.
VEINES-QoL/Sym	It describes the lower limb pain in detail and the QOL part can be applied separately	It does not involve sleep and venous ulcer.
CCVLUQ	It is easy to understand, short time-consuming and it has good reliability. So it is suitable for measuring the life quality of patients with venous ulceration of lower limbs.	Few national applications
DVTQOL	Special attention is paid to the anticoagulant medication in patients with DVT, which is very suitable for the measurement of patients with DVT.	It has few national applications and has no official Chinese version yet.
VLU-QoL	Comprehensive coverage	It has plenty of items
SPVLU-5D	Aim at patients with venous ulceration of lower limb.	Seldom applied
CIVIQ-14	It is more concise, convenient and stable, compared to CIVIQ-20.	It does not involve ulcer

## 4 Discussion

### 4.1 Analysis of the basic situation of the QoL in patients with CVD

Ten scales covering various QoL aspects in patients with CVD were included in the study, and their basic information, psychometric indicators, dimension content, scoring methods, and advantages and disadvantages were sorted out and compared. According to the scope of application, the scales could be divided into universal and specific scales. Universal scales, such as SF-36 and EQ-5D, are widely used, covering mainly pain, daily life, physical activity, and emotional assessment, and the assessment results are suitable for comparison with the QoL aspects of patients with different diseases.^[18,19]^ However, although the scope of application of specific scales is narrower, its more targeted advantages are more apparent, involving problems not included in the universal scale. For example, AVVQ and CCVLUQ will form a dimension due to the change in the appearance of lower limbs caused by diseases.^[20]^ The Deep Venous Thrombosis Quality of Life (DVTQOL) questionnaire also forms a single dimension for patients’ anticoagulation management, which better reflects the real causes of patients’ QoL deterioration and provides researchers with ideas to further their research.^[21,22]^

### 4.2 CVD QoL scale should be selected more specifically

Before research on the QoL in patients with CVD, it is necessary to understand and evaluate the various scales as much as possible, carefully select the appropriate scale, and consider the differences and scope of application of each scale. The involved domains of different rating scales were summarized in Table 4, and we could find that AVVQ is the only scale that specified at the conditions of the CVD, involving patients’ feelings, appearance, function status, complications, and ulcerations.^[23]^ Other scales focused more on the whole-body reaction, for instance, sleep, emotion, and diet. Therefore, selection of scales should comply to the study aim according to different domains. The advantages and disadvantages of each scale mentioned in this review are reported in Table 5. Such aspects should be considered by future researchers when choosing a scale. The author suggests that the reference standard for selecting the CVD QoL scale should be divided into 2 parts:

(1) according to the research object: in addition to the scales included in this study, the Chronic Venous Insufficiency Questionnaire—14 Item (CIVIQ-14), the ChronIc Venous Insufficiency Questionnaire—20 Item (CIVIQ-20), and the Venous Insufficiency Epidemiological and Economic Studies (VEINES) questionnaire, divided into 2 subscales; symptoms (VEINES-Sym) and QoL (VEINES-QoL), are scales developed specifically for patients with varicose veins, DVTQOL is for patients with deep vein thrombosis, and CCVLUQ, VLU-QoL, and SPVLU-5D are specific questionnaires for patients with venous ulcers.^[24,25]^ All the scales make the research outcomes more representative of the characteristics of the disease.
(2) According to the research purpose and measurement content selection: due to the differences in the criteria assessed among the scales, if the researcher seeks to understand the appearance of lesions in patients with CVDs, it is recommended to choose AVVQ, CIVIQ-20, and other scales with related content. In addition, the number of scale entries and the time spent on evaluation should be considered in the study design. QoL is commonly an outcome variable and will be combined with the evaluation of other contents. If the evaluation content is too much and takes a long time, it may lead to decreased patient cooperation, thus hindering research work and affecting the quality of the research. Therefore, under the premise of ensuring the research objective and measurement content, selecting a scale with a reasonable evaluation time is also key to the research design.

## 5 Limitations

Our review had several limitations to address when interpreting our results. First, our study was a narrative review and did not perform a quantitative comparison of CVD scales. Second, current scales of CVD QoL are subject to limitations, which were summarized in our review; hence, specific attention should be paid during application. Third, there was a lack of scale that can be easily used in outpatient settings.

## 6 Conclusion

This study summarized the dimensions, scoring methods, and psychometric indicators of the 10 most commonly used CVD QoL assessment scales in the literature, and thereby help researchers to assess the QoL in patients with CVDs. As the current scales have different specific implications, the researcher should carefully select appropriate scales before conducting their research so that the results of the QoL in patients with CVD are scientifically robust and reliable.

## Author contributions

**Conceptualization:** Zhoupeng Wu, Yukui Ma.

**Data curation:** Yukui Ma.

**Formal analysis:** Zhoupeng Wu.

**Investigation:** Zhoupeng Wu, Yukui Ma.

**Methodology:** Zhoupeng Wu.

**Supervision:** Yukui Ma.

**Validation:** Yukui Ma.

**Writing – original draft:** Zhoupeng Wu.

**Writing – review & editing:** Yukui Ma.
